# Analysis of perioperative autonomic nervous system activity to visualize stress in pediatric patients undergoing alveolar bone graft surgery

**DOI:** 10.1007/s10877-024-01210-w

**Published:** 2024-08-22

**Authors:** Akari Uto, Kaoru Yamashita, Shusei Yoshimine, Minako Uchino, Toshiro Kibe, Mitsutaka Sugimura

**Affiliations:** 1 Department of Dental Anesthesiology, Field of Oral and Maxillofacial Rehabilitation, Advanced Therapeutics Course, Graduate School of Medical and Dental Sciences, Kagoshima University, Kagoshima, Japan; 2 Department of Oral and Maxillofacial Surgery, Field of Oral and Maxillofacial Rehabilitation, Advanced Therapeutics Course, Graduate School of Medical and Dental Sciences, Kagoshima University, Kagoshima, Japan

**Keywords:** Autonomic nervous system activity, Alveolar bone grafting, General anesthesia, Perioperative stress, Pediatric patients

## Abstract

Perioperative stress in pediatric patients is often difficult to assess via interviews; thus, an objective measure to assess perioperative stress is needed. To visualize perioperative stress, we observed autonomic nervous system (ANS) activity, circulatory dynamics, and psychological status in pediatric patients undergoing alveolar bone grafting under general anesthesia. This prospective observational study included 40 patients aged 8–12 years who were scheduled for alveolar bone grafting in our hospital. ANS activity was analyzed using heart rate variability the day before surgery, during general anesthesia, 2 h postoperatively, 24 h postoperatively, and the day before discharge. ANS assessment included LF/HF (sympathetic nervous system activity) and HF (parasympathetic nervous system activity). Additionally, heart rate (HR), systolic blood pressure (SBP), face scale (FS) score were recorded. Data from 31 patients, excluding dropouts, were analyzed. The ratio of change to the preoperative value was compared. After surgery, the LF/HF, HR, SBP, and FS score significantly increased (*P* < 0.01) and HF significantly decreased (2 h postoperatively: *P* < 0.05, 24 h postoperatively, before discharge: *P* < 0.01). SBP recovered to preoperative values 24 h postoperatively, and HR and FS scores recovered to preoperative values before discharge. However, even before discharge, LF/HF remained significantly higher than preoperative values, and HF remained significantly lower than preoperative values (*P* < 0.01). **Conclusion** We observed perioperative stress from multiple perspectives. Circulatory dynamics and psychological status recovered by the day before discharge; however, ANS activity did not. Therefore, evaluating ANS activity may be useful in visualizing potential perioperative stress in pediatric patients.

## Introduction

Surgery under general anesthesia is stressful for pediatric patients [1]. In addition to the stress caused by surgical invasion and environmental changes resulting from hospitalization, patients may develop complications such as nausea, vomiting, and pain [2, 3]. For example, alveolar bone grafting, which is commonly performed in pediatric patients with a history of cleft lip and palate, often involves grafting of the cancellous bone from the anterior iliac crest into the maxillary alveolar cleft under general anesthesia [4, 5]; it may result in postoperative pain and swelling of the maxilla and the iliac donor site. Moreover, gait disturbance has been reported for an average of 6.6 days postoperatively [6]. In surgeries that have perioperative stress as a concern, the stress of the patient throughout the perioperative period should be visualized and understood.

In general, interviews, questionnaires and circulatory dynamics are used to assess perioperative stress, but pediatric patients often find it more difficult to express their condition than adults [7]. Additionally, self-report tools, such as face scale (FS) scores, have limitations related to children’s comprehension and communication skills [8]. Behavioral observation methods can introduce variability between assessors.　In addition, blood sampling is considered invasive and stressful for children, and is not a suitable method for stress assessment in pediatric patients. Thus, it is often difficult to visualize and understand the extent of perioperative stress in pediatric patients; therefore, a non-invasive and objective method to monitor children’s stress should be established.

Stress is associated with autonomic nervous system (ANS) activity [8, 9]. There are several methods for analyzing ANS activity, and heart rate variability (HRV) analysis using electrocardiogram (ECG) data enables monitoring ANS activity without the requirement for invasive procedures in patients [8, 10–16]. Although changes in ANS activity due to general anesthesia have been discussed in previous reports [17, 18], no reports have evaluated ANS activity during the perioperative period in pediatric patients who underwent uniform surgical procedures, and there are many unclear points about perioperative ANS activity in pediatric patients undergoing surgery under general anesthesia.

In this study, in addition to circulatory dynamics and psychological status, which have been used as indicators for stress assessment, we used HRV analysis to observe changes in ANS activity in pediatric patients undergoing alveolar bone graft surgery under general anesthesia. We hypothesized that this observation would enable us to visualize the perioperative stress and analyzed changes in ANS activity, circulatory dynamics, and psychological status during the perioperative period.

## Methods

### Study design/sample

This prospective observational study was approved by the Kagoshima University Hospital Ethics Review Committee (approval no. 190086) and adhered to the tenets of the Declaration of Helsinki. The study was explained to the parents, and age-appropriate explanations were provided to the participants. Written informed consent for study participation was obtained from the parents. This study included patients aged 8–12 years with a diagnosis of cleft lip and palate who visited the Department of Oral and Maxillofacial Surgery at the Kagoshima University Hospital and were scheduled to undergo alveolar bone grafting under general anesthesia with sevoflurane at the same hospital between October 2019 and February 2022. All patients were admitted the day before surgery, and data acquisition was performed during hospitalization. The following patients were excluded: patients with conditions affecting the ANS, those receiving regular medications, those with a history of drug allergy, those with psychosis or psychiatric symptoms that would make study participation difficult, and those who were otherwise deemed ineligible by the dentist. Patients were permitted to eat up to 8.5 h before surgery, drink clear liquids up to 2 h before surgery, and, then discontinue all intake 2 h before surgery.

### Data collection environment and protocol

**Fig. 1:**
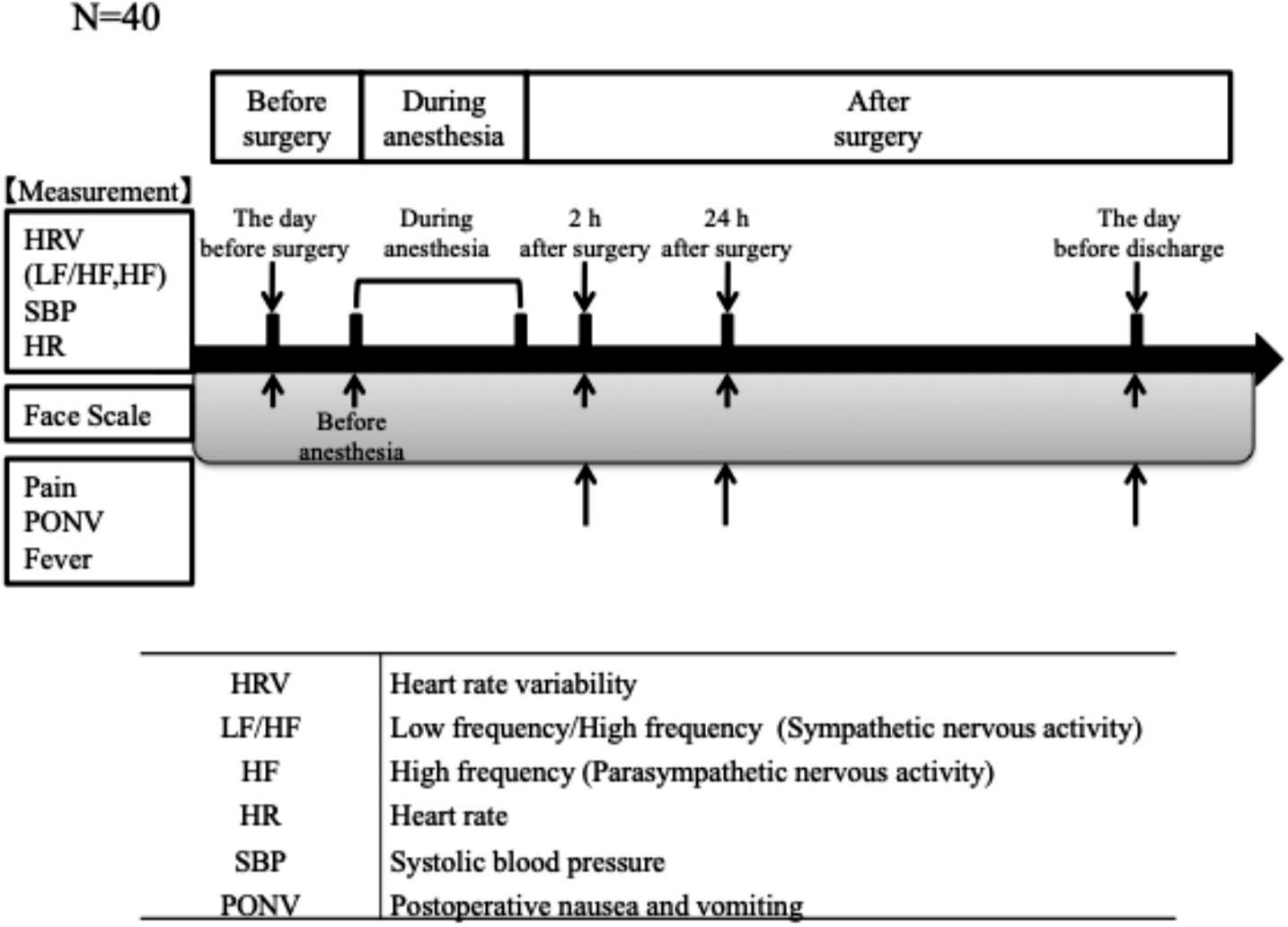
Time course of assessments. Data were measured preoperatively, during general anesthesia, 2 h postoperatively, 24 h postoperatively, and before discharge. Abbreviations: HRV, heart rate variability; LF, low frequency; HF, high frequency; SBP, systolic blood pressure; HR, heart rate; PONV, postoperative nausea and vomiting

In this study, ANS and circulatory dynamics were evaluated in the supine position, either on the operating bed or on the hospital bed at rest. ANS and circulatory dynamics were evaluated the day before surgery, during general anesthesia, 2 h postoperatively, 24 h postoperatively, and before discharge (7 days postoperatively) (Fig. 1). ANS activity was recorded the day before surgery, 2 h postoperatively, 24 h postoperatively, and the day before discharge (postoperative day 7) in the hospital room for 5 min and from the beginning to the end of anesthesia in the operating room. The measurement duration in the hospital room adhered to HRV guidelines [19]. To evaluate circulatory dynamics, heart rate (HR) and systolic blood pressure (SBP) were recorded. When measuring postoperative ANS activity, we also evaluated pain, postoperative nausea and vomiting (PONV), and fever. Face scale (FS) scores were recorded the day before surgery, before anesthesia, and at the time of postoperative ANS activity measurement.

### HRV

ANS activity was assessed via HRV analysis using three-point trigger ECG data. ECG data were acquired using the MWM01 ECG monitor (GMS, Tokyo, Japan) and analyzed using Memcalc-Mackin2 (GMS). For this study, we employed the frequency-domain analysis method, which is preferred for short-term recordings of ANS activity [19]. The high frequency (HF) (0.15–0.4 Hz; mediated by the parasympathetic nervous system) and low frequency (LF) (0.04–0.15 Hz; mediated by the parasympathetic and sympathetic nervous system) components were analyzed to quantify the activities of the sympathetic and parasympathetic nervous systems from the HRV parameters obtained from the ECG data. The evaluation items were LF/HF (the parameter of sympathetic nervous system activity) and HF (the parameter of parasympathetic nervous system activity) [13, 20, 21].

### FS score evaluation

Since most patients were aged around 10 years and could find it difficult to express pain and their psychological state to the interviewer, the FS was used with HRV measurements (Fig. 1). As an evaluation method using the FS, we asked the patients to select the picture that most closely represented their feelings from six pictures presenting very happy to very unhappy facial expressions. The happiest facial expression was evaluated as 1 point, and the saddest facial expression was evaluated as 6 points.

### Anesthesia

Anesthesia was administered via slow induction with 5% sevoflurane or rapid induction with propofol 1–2 mg/kg. Acetated Ringer’s solution with 1% glucose fluid replacement and remifentanil 0.3 µg/kg/min were administered after intravenous catheterization. Rocuronium 0.8 mg/kg was administered, and oral intubation was performed using a Macintosh laryngoscope. Anesthesia was maintained with 1.5–2% sevoflurane and continuous intraoperative remifentanil at a dose of 0.1–0.5 µg/kg/min. As a local anesthetic, 0.5% lidocaine containing 1:200,000 epinephrine was administered by the surgeon in the alveolar cleft region and iliac donor site before the start of surgery. Postoperative analgesia involved intravenous administration of 15 mg/kg acetaminophen over 15 min when the surgeon began suturing the iliac region, 1 mg/kg flurbiprofen axetil over 5 min after all sutures were completed, and 3 mg/kg (upper limit 75 mg) of a local anesthetic (ropivacaine hydrochloride hydrate) at the iliac donor site at the end of suturing.

### Operative procedure

Cancellous bone was grafted from the anterior iliac crest into the maxillary alveolar cleft in all cases. The surgery was performed by the same team, including an oral surgeon, using the procedure reported by Boyne and Sands [22]. The bone harvesting technique was based on the report by Robertson and Barron [23].

### Postoperative management

From the time the patient returned to the ward until 24 h postoperatively, 15 mg/kg acetaminophen was administered intravenously every 6 h. Ibuprofen was prescribed if the patients experienced pain. Patients undergoing alveolar bone graft surgery at our hospital were instructed to rest in the bed on the first postoperative day. They were allowed to move using a wheelchair from 2 days postoperatively and walk with a walker from 4 days postoperatively. Unassisted walking was allowed 6 days postoperatively, and patients were discharged 8 days postoperatively. Oral splints were provided for wound healing postoperatively.

### Statistical analysis

The required sample size was calculated using a power analysis (α = 0.05; β = 0.2) and set at 40 to account for dropouts. All statistical analyses were performed using GraphPad Prism version 6 (San Diego, CA, USA). Comparisons of changes over time in preoperative values for LF/HF, HF, HR, SBP, and FS scores were performed using the Friedman test and Steel–Dwass test as a post hoc test. The level of statistical significance was set at P values < 0.05.

## Results

Among the 40 eligible patients, nine were excluded because their parents refused to consent to data collection; thus, we could not obtain ECG recordings, interviews, or other data from their hospital rooms. Thus, the data of 31 patients were analyzed. The mean age, weight, and height of the analyzed patients were 9.87 ± 1.07 years, 30.29 ± 5.98 kg, and 134.62 ± 8.17 cm, respectively (Table 1).

**Table 1:** Patient characteristics (*N*=31)

Characteristic	Proportion or mean ± SD
Sex	
Male/Female	17 / 14
Age (years)	9.87 ± 1.07
Weight (kg)	30.29 ± 5.98
Height (cm)	134.62 ± 8.17
Operative duration (min)	140.32 ± 31.71
Anesthesia duration (min)	237.68 ± 36.21
Blood loss (mL)	120.34 ± 97.15
Cleft side	
Left/Right/Both	19 / 8 / 4
Induction	
Slow/Rapid	16 / 15

Abbreviations SD, standard deviation

**Fig. 2:**
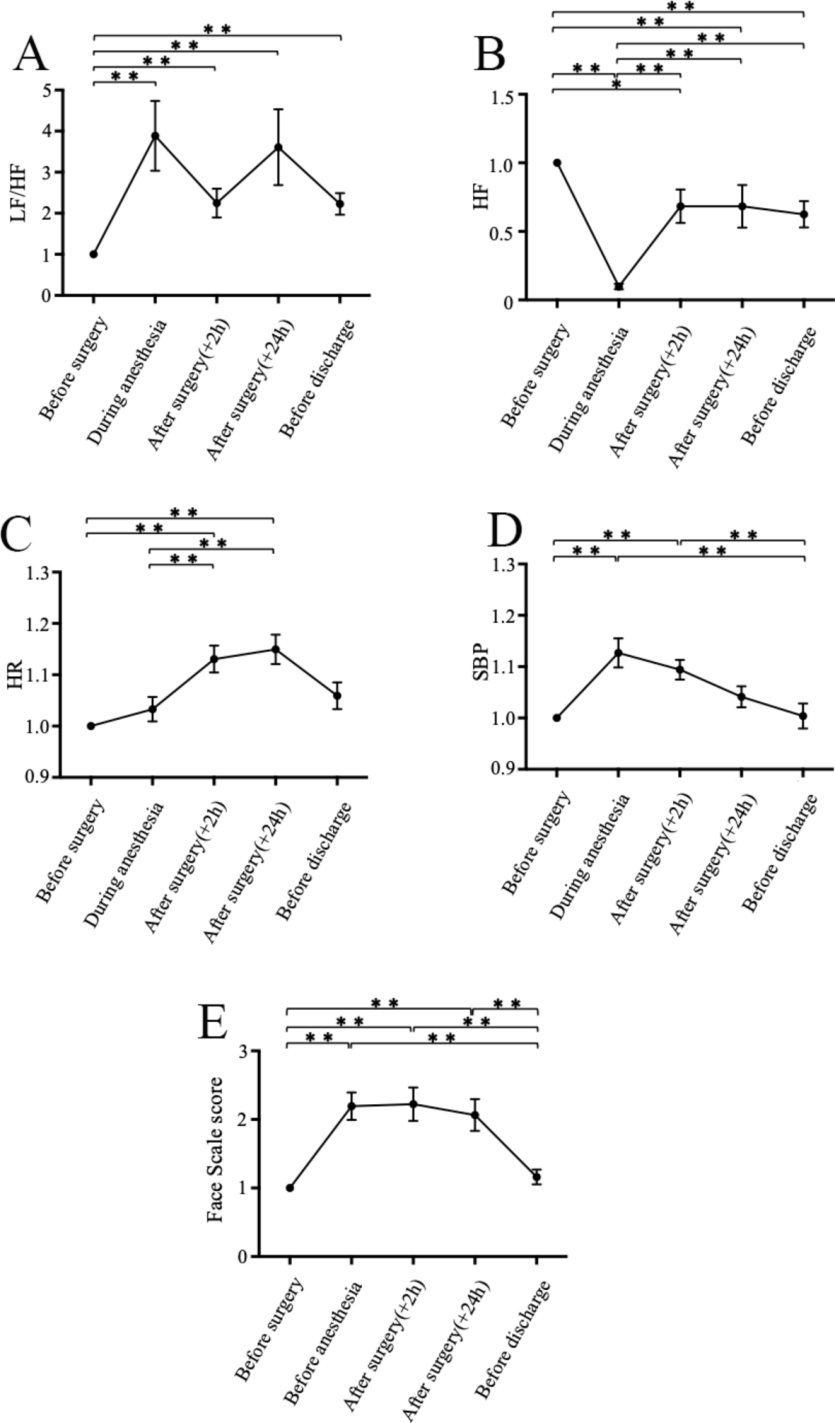
Comparison of (**a**) LF/HF, (**b**) HF, (**c**) HR, (**d**) SBP, and (**e**) face scale score at each time point between the groups. The face scale before anesthesia was assessed after entering the operating room. LF/HF, HF, HR, SBP, and face scale scores are expressed as relative ratios to preoperative values. Abbreviations: LF/HF, low-to-high frequency ratio; HF, high frequency; HR, heart rate; SBP, systolic blood pressure. All data are presented as mean and standard error. The level of statistical significance was set at P values < 0.05. * *P* < 0.05. ** *P* < 0.01

LF/HF, HF, HR, SBP, and FS scores were presented as relative ratios to preoperative values (Fig. 2).

LF/HF during general anesthesia (3.89 ± 0.85) was significantly higher than the preoperative value (*P* < 0.01). Thereafter, LF/HF remained significantly higher at 2 h postoperatively (2.25 ± 0.35, *P* < 0.01), 24 h postoperatively (3.61 ± 0.92, *P* < 0.01), and the day before discharge (2.23 ± 0.26, *P* < 0.01) than the preoperative values (Fig. 2a).

HF significantly decreased from the day before surgery to during general anesthesia (0.10 ± 0.02, *P* < 0.01). The values 2 h postoperatively (0.68 ± 0.12), 24 h postoperatively (0.68 ± 0.16), and before discharge (0.63 ± 0.10) were significantly higher than the value during general anesthesia (*P* < 0.01), but remained significantly lower than the preoperative value (2 h postoperatively: *P* < 0.05, 24 h postoperatively: *P* < 0.01, before discharge: *P* < 0.01) (Fig. 2b).

HR during general anesthesia (1.03 ± 0.02) was not significantly different from the preoperative value. However, it significantly increased 2 h postoperatively (1.13 ± 0.03, *P* < 0.01) and 24 h postoperatively (1.15 ± 0.03, *P* < 0.01) compared with the preoperative value. HR on the day before discharge (1.06 ± 0.0.03) was not significantly different from the preoperative value (Fig. 2c).

SBP increased significantly during general anesthesia (1.13 ± 0.03, *P* < 0.01) and 2 h postoperatively (1.09 ± 0.02, *P* < 0.01) compared with the preoperative value; however, SBP values 24 h postoperatively (1.04 ± 0.03) and on the day before discharge (1.00 ± 0.02) were not significantly different from the preoperative value (Fig. 2d).

FS scores before anesthesia (2.19 ± 0.2), 2 h postoperatively (2.22 ± 0.24), and 24 h postoperatively (2.07 ± 0.23) were significantly higher than the preoperative value (*P* < 0.01). However, the value on the day before discharge (1.16 ± 0.11) was not significantly different from the preoperative value (Fig. 2e).

Table 2 presents the results of pain, PONV, and fever among the patients at different time points. Patients were most likely to complain of pain and fever at 24 h postoperatively, with only a few patients having persistent pain, PONV, and fever until the day before discharge.

**Table 2:** Interview results of pain, PONV, and fever among patients at different time points (*N* = 31)

	2 h after surgery	24 h after surgery	Day before discharge
Pain (+)^†^	8	21	4
Pain (-)^‡^	23	10	27
PONV (+)	2	3	0
PONV (-)	29	28	31
Fever (+)	2	13	1
Fever (-)	29	18	30

^**†**^(+) presence; ^**‡**^(-) absence

Abbreviations: PONV, postoperative nausea and vomiting

The threshold for the presence or absence of fever was 37.5℃

## Discussion

In this study, ANS activity was observed with uniform surgical procedures and perioperative management in order to visualize perioperative stress in pediatric patients. In addition to vital signs and interviews, which are commonly used to assess stress, ANS activity assessment was also used to evaluate perioperative stress from multiple perspectives. As a result, after alveolar bone graft surgery under general anesthesia, HR, SBP, and FS score were significantly increased compared to preoperative values. Regarding ANS activity, LF/HF significantly increased and HF significantly decreased compared to preoperative values. Significant increases in FS scores and either circulatory dynamics (SBP or HR) were observed compared to preoperative values during general anesthesia, 2 h postoperatively, and 24 h postoperatively. These results suggest the existence of perioperative stress after surgery under general anesthesia. Furthermore, at that time, LF/HF increased and HF decreased, and the ANS activity also supported the existence of perioperative stress. On the other hand, before discharge, LF/HF increased and HF decreased, but other indicators such as SBP, HR, and FS score had recovered to preoperative values. This suggests that ANS activity indicators may be useful for monitoring potential stress, which is difficult to assess using vital signs or interviews.

This study revealed that fluctuations in the ANS activity after alveolar bone grafting under general anesthesia persisted until the day before discharge, which was 7 days postoperatively. The ANS activity after general anesthesia has been studied in several reports [18, 24]. However, there are no reports evaluating long-term ANS activity in pediatric patients undergoing the same surgical procedure. Therefore, we evaluated ANS activity over time based on previous studies [9, 25] and limited the target population to pediatric patients undergoing alveolar bone grafting. Previous studies reported that general anesthesia decreases HRV parameters [18, 19, 24, 26], and surgical invasion or stress increases sympathetic nervous system activity (LF/HF) and decreases parasympathetic nervous system activity (HF), resulting in an imbalance in ANS activity [27, 28]. In this study, the sympathetic nervous system activity increased, whereas the parasympathetic nervous system activity decreased from the preoperative period during anesthesia. This may be because of the suppression of ANS activity by anesthetics and the increase in sympathetic nervous system activity due to various stimuli, such as tracheal intubation and surgical invasion [12].

Previous studies examining changes in HRV indices following surgery under general anesthesia showed that ANS activity gradually returned to baseline over a period of 2 to 8 h postoperatively in adults [29] and within 24 h postoperatively in children younger than 8 years. [24] In our study, wherein patients aged 8–12 years underwent alveolar bone grafting, the ANS activity (LF/HF and HF) on the day before discharge were both significantly different from the preoperative values, indicating that fluctuations in ANS activity due to surgery under general anesthesia persisted until the day before discharge. On the other hand, SBP returned to preoperative values 24 h postoperatively, and HR and FS score returned to preoperative values on the day before discharge. Few patients reported pain before discharge, and none of the patients reported PONV.

In a previous study on stress assessment, stress evaluation of on-duty anesthesiologists was conducted using HRV analysis and psychological tests. The results showed that the ANS activity was suppressed under stress; however, the results of the questionnaire assessment remained unchanged. Therefore, it has been reported that HRV analysis may be useful for potential stress monitoring [30]. Similarly, in this study, only fluctuations in the ANS activity (LF/HF and HF) persisted until before discharge. This suggests that analysis of ANS activity may be useful in visualizing perioperative stress in pediatric patients, which is difficult to assess via an interview or vital signs.

It has been reported that fluctuations in the ANS activity are associated with stress [9, 10]. Inpatients are exposed to a variety of stresses, including unfamiliar environments, loss of independence, threats from their disease, and complications such as pain and PONV [31]. Furthermore, at our hospital, all patients who undergo alveolar bone grafting are instructed bed rest for 2 days postoperatively and to wear an oral splint for wound healing. Moreover, they are not allowed to walk independently for at least 6 days. According to previous studies on alveolar bone grafting at other institutions, gait disturbance persists for an average of 6.6 days [6]. Patients were able to resume school from an average of 12.6 days postoperatively, and sports activities were resumed from 1 month postoperatively [4]. Thus, alveolar bone grafting under general anesthesia may impose long-term physical limitations and emotional distress on patients, which may cause ANS fluctuations.

Previous reports have shown that music listening and video games can effectively reduce stress in children undergoing general anesthesia and surgery [1]. We propose that these methods may be effective in improving perioperative stress in pediatric patients. Additionally, regular ANS monitoring throughout the perioperative period might allow early identification of stress and interventions, resulting in improved patient satisfaction during hospitalization.

The strength of this study is that we were able to observe perioperative ANS activity in pediatric patients over 1 week, with uniform surgical procedures and perioperative management. Another advantage was that we were able to assess perioperative stress from multiple perspectives by evaluating ANS activity as well as circulatory dynamics and psychological status. However, one limitation of this study was that ECG recordings were unavailable after the day of discharge, making it impossible to assess whether the ANS activity subsequently recovered to the preoperative levels. Furthermore, while HRV is used to assess ANS activity, it is influenced by factors, such as age, measurement time, environment, pain, and anxiety [9, 14, 27]. The purpose of this study was to observe the changes in ANS activity resulting from these various factors, therefore the direct association between individual factors and HRV was not examined.

In conclusion, the present study showed that fluctuations in vital signs, psychological status, and ANS activity were observed after alveolar bone graft surgery under general anesthesia in pediatric patients, and the results of ANS activity analysis also suggested the presence of perioperative stress. However, on the day before discharge, although vital signs and psychological state had recovered to preoperative states, fluctuations in ANS activity persisted. Only ANS activity did not recover by the day of discharge, suggesting that ANS activity can be used to assess stress not captured by other measures and may lead to improved quality of perioperative management. If a specific method for monitoring ANS activity is established, analysis of ANS activity may be useful in visualizing perioperative stress in pediatric patients, which is difficult to assess via an interview or vital signs. The relationship between ANS activity and perioperative stress requires further investigation.

## Data Availability

No datasets were generated or analysed during the current study.
